# Evaluation of Droplet Digital Polymerase Chain Reaction (ddPCR) for the Absolute Quantification of *Aspergillus* species in the Human Airway

**DOI:** 10.3390/ijms21093043

**Published:** 2020-04-26

**Authors:** Tuang Yeow Poh, Nur A’tikah Binte Mohamed Ali, Louisa L.Y. Chan, Pei Yee Tiew, Sanjay H. Chotirmall

**Affiliations:** 1 Translational Respiratory Research Laboratory, Lee Kong Chian School of Medicine, Nanyang Technological University, 11 Mandalay Road, Singapore 308232, Singapore; 2 Department of Respiratory and Critical Care Medicine, Singapore General Hospital, Singapore 169608, Singapore

**Keywords:** *Aspergillus fumigatus*, *Aspergillus terreus*, qPCR, ddPCR, quantification

## Abstract

Background: Prior studies illustrate the presence and clinical importance of detecting *Aspergillus* species in the airways of patients with chronic respiratory disease. Despite this, a low fungal biomass and the presence of PCR inhibitors limits the usefulness of quantitative PCR (qPCR) for accurate absolute quantification of *Aspergillus* in specimens from the human airway. Droplet digital PCR (ddPCR) however, presents an alternative methodology allowing higher sensitivity and accuracy of such quantification but remains to be evaluated in head-to-head fashion using specimens from the human airway. Here, we implement a standard duplex TaqMan PCR protocol, and assess if ddPCR is superior in quantifying airway *Aspergillus* when compared to standard qPCR. Methods: The molecular approaches of qPCR and ddPCR were applied to DNA fungal extracts in *n* = 20 sputum specimens obtained from non-diseased (*n* = 4), chronic obstructive pulmonary disease (COPD; *n* = 8) and non-cystic fibrosis bronchiectasis (*n* = 8) patients where *Aspergillus* status was known. DNA was extracted and qPCR and ddPCR performed on all specimens with appropriate controls and head-to-head comparisons performed. Results: Standard qPCR and ddPCR were both able to detect, even at low abundance, *Aspergillus* species (*Aspergillus fumigatus - A. fumigatus* and *Aspergillus terreus - A. terreus*) from specimens known to contain the respective fungi. Importantly, however, ddPCR was superior for the detection of *A. terreus* particularly when present at very low abundance and demonstrates greater resistance to PCR inhibition compared to qPCR. Conclusion: ddPCR has greater sensitivity for *A. terreus* detection from respiratory specimens, and is more resistant to PCR inhibition, important attributes considering the importance of *A. terreus* species in chronic respiratory disease states such as bronchiectasis.

## 1. Introduction

Quantitative PCR (qPCR) is an adaptation of standard PCR permitting the detection and real-time quantification of specific target amplification products. A target DNA sequence is selectively amplified using sequence-specific primers, a reporter and quencher labeled dual fluorochrome, an oligonucleotide hybridization probe and a Taq DNA polymerase enzyme. During amplification, the probe specifically hybridizes the accumulating product, and the endonuclease activity of the Taq DNA polymerase cleaves reporter-labeled nucleotides resulting in detectable fluorescence. Reactions are characterized by the time duration during the standard 40 cycles of qPCR amplification where a threshold of baseline fluorescence (C_qs_) is exceeded. TaqMan qPCR is established as a useful method for the detection and identification of *Aspergillus* species in clinical samples including the airway [1,2,3,4,5]. Using these and other next-generation sequencing approaches, our group has demonstrated high levels of airway *Aspergillus* in patients with bronchiectasis where higher qPCR-derived *A. fumigatus* and *A. terreus* is associated with poorer clinical outcome [1,3,6]. Importantly, however, to determine absolute quantification of *Aspergillus* 18S rRNA, a serial dilution of *plasmid containing 18S* DNA is necessary for the generation of a standard curve on each plate, a time consuming and costly process limiting the specimens that can be studied. In addition, optimization of the employed standard curves is required, which in itself, demonstrates dynamic and differing ranges for the absolute quantification of *Aspergillus* species [7]. The results of even standard and test specimens may vary based on reaction efficiencies and differences in specimen content including the presence of inhibitors [8,9]. For all these reasons, an improved and alternative method may be beneficial.

**Figure 1:**
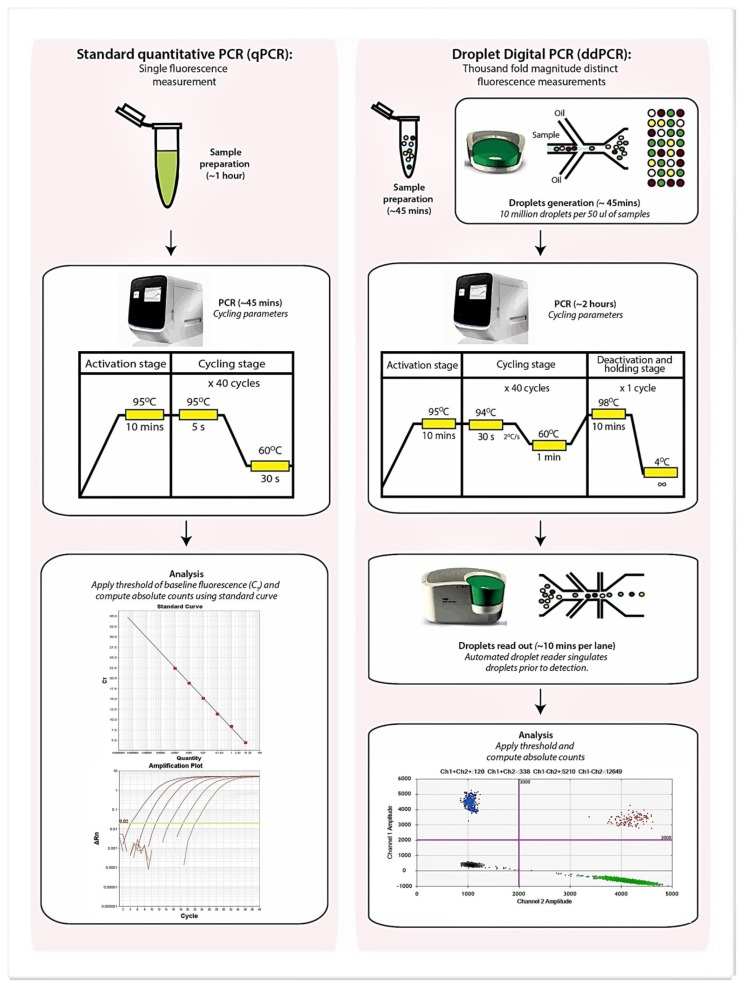
Schematic diagram illustrating protocol related differences between qPCR and droplet digital PCR (ddPCR) including the estimated time required at each step. Sample preparation for both qPCR and ddPCR while comparable is slightly longer for qPCR due to a requirement for standard curve preparation to allow quantification and inclusion of an internal positive control to exclude PCR inhibition.

Recently, droplet digital PCR (ddPCR) has been developed and could potentially circumvent issues associated with qPCR [10,11,12]. This technique, based on partitioning the PCR reaction mix into a thousand-fold magnitudes smaller and segregated reaction droplets allows amplification of the respective target(s) within each individual droplet which is then quantified by a target-dependent fluorescence signal (Figure 1). The digital aspect of this approach relies on distributing the target gene into a significant number of partitions (or droplets) such that each receives a number of genes (i.e., 0, 1, 2, etc.). Performing PCR on such partitions results in the amplification being labeled positive (in those containing the target) or negative (no amplification). As positive readouts potentially contain more than a single gene copy of the target molecule, a simple summing of the number of positives will not yield the correct number of target molecules that may be present. Therefore, Poisson statistics are applied in ddPCR to estimate the total number of target molecules present within an interrogated specimen and avoids the need for reference to a standard curve [10,11,12]. As ddPCR represents an end-point PCR reaction, data are unaffected by variations to reaction efficiency and the absolute copy number of the target genes can be determined with confidence so long as the fluorescence readout is correctly partitioned to positive and negative droplets. The high precision and accuracy of ddPCR further reduces the need for technical replicates which improves experimental throughput, saves time, and effectively permits accurate quantification of targets in low volume human specimens such as that from the airway [10,11,12]. Table 1 summarizes the comparisons between qPCR and ddPCR.

In this study, we evaluate the accuracy and sensitivity of ddPCR (as compared to qPCR) in detecting *Aspergillus* species in the normal and diseased airway from respiratory specimens. To allow concurrent detection of *A. fumigatus* and *A. terreus* in our samples, we developed duplex primers and probes that allow interrogation of both species by ddPCR.

## 2. Results

### 2.1. Assessment for the Specificity of the A. fumigatus and A. terreus Duplex TaqMan Primer and Probe Set Used for ddPCR Evaluation

**Figure 2:**
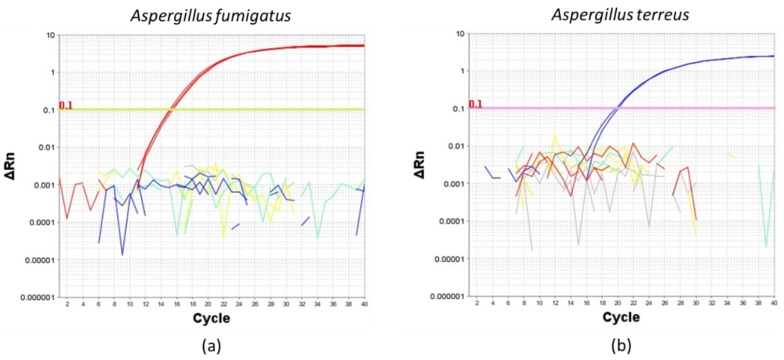
Amplification plots illustrating the specificity of (**a**) *Aspergillus fumigatus* and (**b**) *Aspergillus terreus* TaqMan primers and probes using a reaction mix containing 0.1 ng of extracted DNA from pure cultures of (**a**) *Aspergillus fumigatus* (red line), (**b**) *Aspergillus terreus* (blue line), *Aspergillus* flavus (yellow line) and *Aspergillus niger* (green line). No template control is indicated by gray lines. ∆Rn: delta normalized reported value.

To determine the specificity of the TaqMan primers and probes, we first amplified *A. fumigatus* or *A.terreus* from conidial DNA extracted from four common *Aspergillus* species isolated from the human airway, namely *A. fumigatus*, *A. terreus*, *A. flavus* and *A. niger* (Figure 2). Importantly, *A. fumigatus* and *A. terreus* TaqMan primers and probes only amplified their respective DNA extracts and not extracts obtained from other species confirming the specificity for the primers and probes to be used for the evaluation of ddPCR (Figure 2).

### 2.2. Evaluation of Limits for the Detection and Quantification of A. fumigatus and A. terreus Using TaqMan Duplex Sets by qPCR and ddPCR

**Figure 3:**
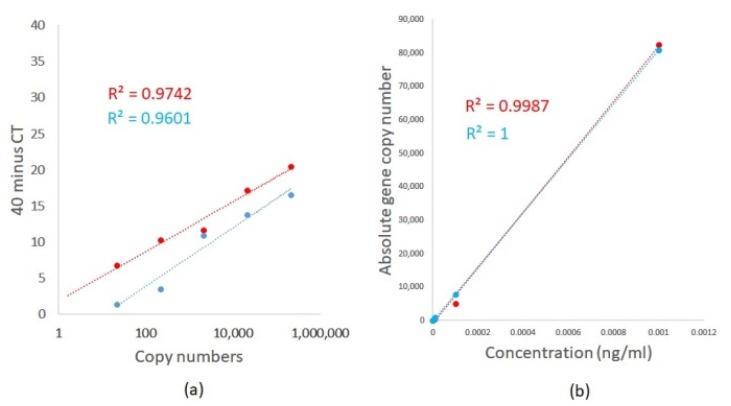
Limit of detection and absolute quantification of *A. fumigatus* (red line) and *A. terreus* (blue line) using (**a**) TaqMan qPCR and (**b**) ddPCR. 18S ITS DNA sequences of *A. fumigatus* and *A. terreus* were cloned into a TOPO plasmid vector and qPCR and ddPCR performed using 10-fold serial dilutions of plasmid from 1 pg/mL to 0.1 fg/mL. Absolute copy numbers are computed by using known sequence and plasmid concentrations (for TaqMan qPCR) and by identifying negative and positive droplets based on a Poisson distribution statistical algorithm (for ddPCR).

The lower limit for the detection and quantification of *A. fumigatus* and *A. terreus* was determined using TOPO plasmids containing the 18S ITS DNA sequence of each fungal species. Plasmid copy number was calculated based on plasmid concentration and sequence using the DNA/RNA copy number calculator (http://endmemo.com/bio/dnacopynum.php). TaqMan qPCR was first performed using a 10-fold serially diluted *Aspergillus* species 18S ITS DNA plasmid from 1 pg/mL to 0.1 fg/mL. Table 2 illustrates the results. We detected femtogram concentrations of plasmid DNA with an R^2^ of 0.9742 and 0.9601, respectively, for *A. fumigatus* and *A. terreus* using TaqMan qPCR (Figure 3a). *Aspergillus* TaqMan qPCR demonstrates amplification efficiencies of 102% and 96.5%, respectively, for *A. fumigatus* and *A. terreus* with a slope of 3.274 and 3.409, respectively (Figure 3a) [14]. Importantly, differing efficiencies between the respective primer and probe sets were observed (Figure 3a). For comparisons and utilizing the same primer and probe sets, ddPCR was similarly able to detect *A. fumigatus* and *A. terreus,* respectively, down to femtogram concentrations of plasmid DNA with superior linear regression R^2^ of 0.9987 and 1, respectively, for *A. fumigatus* and *A. terreus* (Figure 3b). For ddPCR assessment, absolute copy numbers were determined using negative and positive droplets based on a Poisson distribution statistical algorithm without the need for a standard curve (Table 3). Differences were obtained in derived fungal DNA copy numbers between qPCR and ddPCR, whereby qPCR illustrates significantly higher copy numbers (Table 2 and Table 3). Although both approaches have high sensitivity for *A. fumigatus* and *A. terreus* detection, a qPCR-based approach likely overestimates absolute fungal burden in comparison to ddPCR as DNA concentrations are estimated based on spectrophotometer measurements.

### 2.3. Quantification of Airway A. fumigatus and A. terreus in Respiratory Specimens Obtained from Non-Diseased (Healthy) Individuals and Patients with Chronic Obstructive Pulmonary Disease (COPD) and Bronchiectasis by qPCR and ddPCR

**Figure 4:**
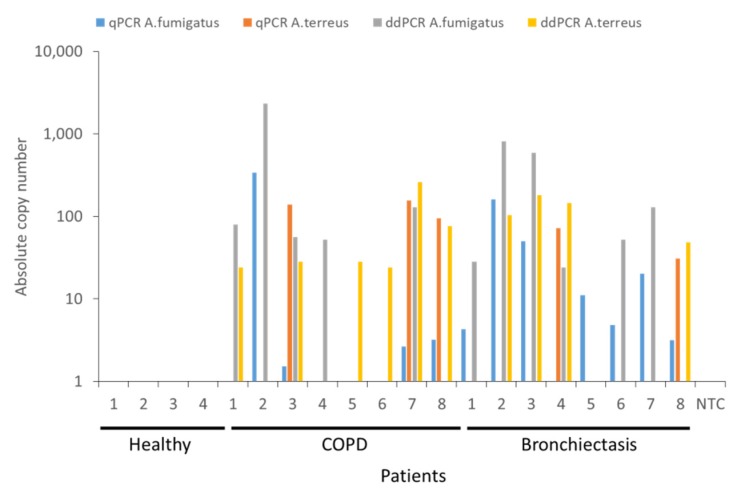
Head-to-head comparison between qPCR and ddPCR for the detection of *A. fumigatus* and *A. terreus* burden in non-diseased (healthy) individuals (*n* = 4) and patients with COPD (*n* = 8) or bronchiectasis (B, *n* = 8). Quantification obtained from TaqMan qPCR is indicated in blue for *A. fumigatus* and orange for *A. terreus,* respectively. Quantification by ddPCR is indicated in gray for *A. fumigatus*) and yellow for *A. terreus,* respectively. NTC: no template control, Asp: *Aspergillus*, qPCR: quantitative polymerase chain reaction, ddPCR: droplet digital PCR, COPD: chronic obstructive pulmonary disease.

Having identified that ddPCR demonstrates greater sensitivity for the detection of *A. fumigatus* and *A. terreus* in earlier experiments, we next evaluated differences in detection and quantification ability using clinical specimens from healthy and diseased individuals. We prospectively recruited *n* = 20 individuals (*n* = 4 non-diseased (healthy), *n* = 8 with COPD and *n* = 8 with bronchiectasis), the latter diseased groups with known and previously detectable *Aspergillus* species based on 18S ITS mycobiome sequencing from our ongoing or previously published works [1,3,15]. While we did not detect any *A. fumigatus* or *A. terreus* in non-diseased (healthy) individuals, all patients with COPD and bronchiectasis demonstrated detectable *A. fumigatus* and/or *A. terreus* in their airway specimens by qPCR and/or ddPCR (Figure 4). All *n* = 4 healthy samples tested were known to be *Aspergillus* negative and the *n* = 16 clinical samples *Aspergillus* positive by 18S ITS sequencing. Using the qPCR and ddPCR approaches, we identified *Aspergillus* species in 13 of the 16 clinical samples (81.3%) by qPCR as opposed to 15 out of the 16 samples (93.8%) by ddPCR. Detection limits by qPCR were determined using known concentrations of plasmid containing *A. fumigatus* or *A. terreus* 18S sequences and ranged between ~2 and 500 copies while copy numbers for ddPCR ranged between 30 and 3000 copies of *A. fumigatus* and/or *A. terreus*, determined by Poisson distribution. No false-positives were detected for the healthy samples and false-positive rates could not be determined for the clinical specimens included as all were known to be *Aspergillus* positive prior to study entry. However, the false-negative rates were evaluated for clinical specimens and found to be 3 out of 16 (18.8%) for qPCR and only a single sample out of 16 (6.3%) for ddPCR. False-negative rates could not be determined for healthy specimens as all were known to be *Aspergillus* negative at study entry. Five of the eight COPD (62.5%) and six of the eight (75%) bronchiectasis patients, respectively, demonstrate *A. fumigatus* by both methods while *A. terreus* was detectable in 75% (*n* = 6) and 50% (*n* = 4) of COPD and bronchiectasis patients, respectively. Importantly, when *A. fumigatus* was considered alone and compared between qPCR and ddPCR approaches, no single approach was superior in its detection (i.e., COPD: four vs. five and bronchiectasis seven vs. six positive by qPCR and ddPCR, respectively) Importantly, for diseased patients demonstrating lowest airway *A. terreus* burdens, ddPCR was better than qPCR in identifying them with greater numbers deemed positive through ddPCR compared with the qPCR approach (Figure 4). ddPCR identified twice as many COPD and bronchiectasis patients as *A. terreus* positive, respectively, when compared with qPCR (i.e., COPD: three vs. six and bronchiectasis two vs. four positives by qPCR and ddPCR, respectively; Figure 4). Therefore, while both qPCR and ddPCR can detect the presence of *A. fumigatus* and *A. terreus* in respiratory specimens comparably, ddPCR demonstrated superiority in the detection of *A. terreus* especially when it was present in ultra-low burden.

### 2.4. Quantification of Airway A. fumigatus by ddPCR is Resistant to PCR Inhibition

**Figure 5:**
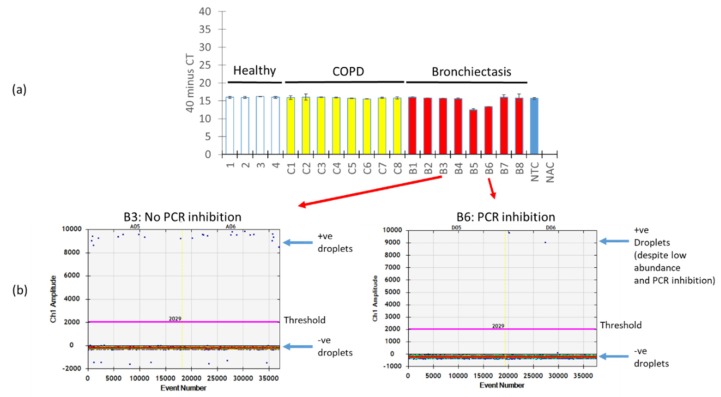
ddPCR is resistant to PCR inhibition. (**a**) Respiratory specimens were “spiked” with an internal positive control and subjected to qPCR illustrating various degrees of PCR inhibition most prominent in specimens B5 and B6. (**b**) One-dimensional amplitude plot from ddPCR illustrating the gating of positive and negative populations (indicated by arrows) that permit an absolute quantification of *A. fumigatus* despite the presence of PCR inhibitors. Thresholds are indicated by a pink line separating positive and negative fractions from ddPCR. +ve: positive, −ve: negative, NTC: no template control, NAC: no amplification control, qPCR: quantitative polymerase chain reaction, ddPCR: droplet digital PCR, COPD: chronic obstructive pulmonary disease, B: bronchiectasis.

A key issue with PCR amplification using biological specimens is the presence of PCR inhibitors which, in turn, affect the quantification of the target PCR product. We next evaluated how the presence of PCR inhibitors in our respiratory specimens affects the quantification of *A. fumigatus* using ddPCR versus qPCR. To assess and identify PCR inhibition across our respiratory specimens, we “spiked in” an internal positive control into the mastermix followed by qPCR. Varying degrees of PCR inhibition across the respiratory specimens were noted, in particular for specimens B5 and B6, where measured absolute counts of *A. fumigatus* would be affected due to the detected C_qs_ differences translating to an almost two-fold change in expression (Figure 5a). Interestingly, however, when we examined two particular specimens: one demonstrating no PCR inhibition (B3) and the other PCR inhibition (B6), ddPCR was resistant to PCR inhibition, as long as segregation is achieved between its negative and positive fractions (Figure 5b).

## 3. Discussion

The study of airway fungal communities, including next-generation sequencing of the pulmonary mycobiome, is gaining clinical and academic interest, particularly in the setting of chronic respiratory disease states such as asthma, COPD, cystic fibrosis and bronchiectasis [1,6,16,17,18,19,20,21,22,23]. Fungi, even in diseased states where dysbiosis is prevalent, demonstrate relatively low abundance at most body sites including the gastrointestinal and respiratory tracts where fungi typically make up less than 5% of the resident microbial community [1,3,24,25,26,27,28,29,30,31]. This low fungal abundance makes 18S ITS targeted amplicon sequencing and qPCR methodological approaches attractive, however, identifying and quantifying fungal load in this manner imposes a lower limit of detection, problematic in cases of ultra-low fungal burdens, and also makes accurate and reliable quantification challenging in the clinical setting. Here, for the first time, we evaluated ddPCR as an alternative to standard qPCR for the detection of *Aspergillus* species from the human airway. While both methods can detect and quantify *Aspergillus* species reliably, ddPCR demonstrates greater sensitivity for *A. terreus* detection and is more resistant to PCR inhibition making it an attractive alternative for the detection of microbes, such as fungi, that occur in low abundance in respiratory specimens but have clinical relevance.

The clinical relevance of low abundance microbes in the airway is well demonstrated in chronic respiratory disease states complicated by the infection such as cystic fibrosis (CF) and bronchiectasis [20,32,33,34,35]. In these settings, the acquisition of a new organism or confirmation that an eradication regime has been effective are clinically critical features for which standard microbiological approaches are lacking. While standard qPCR is useful, it lacks sensitivity below its lower limit of detectable thresholds for individual organisms. In CF and bronchiectasis, the airway ecology is a complex milieu of multiple co-existing organisms from a variety of kingdoms, and hence the ability to detect organisms at ultra-low abundance has value. A clinical example is the identification of allergic bronchopulmonary aspergillosis (ABPA) in CF where likely ultra-low amounts of fungi exist in the airway but are currently either missed or undetected through standard approaches. ddPCR may, therefore, offer an attractive alternative to qPCR in selected clinical settings [20,33,34,35,36,37,38].

One key advantage of ddPCR over traditional TaqMan qPCR is the direct quantification of a target microbe without the need for a standard curve or controls. This leads to improved reproducibility and better accuracy by eliminating reliance on quantitative reference materials whose quantification, source, batch, storage and handling conditions can all influence qPCR results for biological specimens [39]. “Known” quantities of the target, used in TaqMan qPCR standards, as measured by UV spectrophotometry using Nanodrop, employs an indirect quantification approach which potentially affects reliability and accuracy because of quantification assumption uncertainty (i.e., gene copy number per cell and conversions of measured absorption to copy numbers, etc.) [40]. Spectrophotometry quantifies all nucleic acids that absorb at 260 nm which include quantifying DNA and RNA impurities within the standard itself which, when used to quantify the target in samples of unknown quantity, may lead inadvertently to an overestimation.

Our detected higher precision for quantifying a target of ultra-low abundances such as *A. terreus* and the higher run-to-run reproducibility observed with ddPCR is consistent with the binary nature of digital PCR quantification and the findings of others [10,39,40,41,42]. ddPCR quantifies by counting frequencies of positive endpoint PCRs based on a Poisson distribution, whose quantification is not dependent on variability in PCR amplification efficiency, an issue with TaqMan qPCR. Substrate competition and PCR amplification efficiency are likely explanations for the observed variability between qPCR and ddPCR results for *A. fumigatus* and *A. terreus,* respectively, where no superiority of one technique was present for *A. fumigatus* but the latter (ddPCR) better for *A. terreus*. A combined effect of the target number, abundance and amplicon length on reaction mix consumption is also greater for multiplex compared to single-plex reactions. Therefore, if any reaction component is limiting, multiplex reactions can show either significant Cqs delays or even total loss of PCR products particularly for targets of lowest abundance. The improved precision attained through ddPCR is a key consideration for respiratory specimens of ultra-low concentrations or where the target is undetectable by TaqMan qPCR.

Our detected tolerance to PCR inhibitors with ddPCR is consistent with its binary nature [43]. PCR inhibitors function either through DNA sequestration or by reducing PCR amplification efficiency, both of which increase C_qs_ values and lead to underestimation with a TaqMan qPCR approach. Despite reduced amplification (i.e., lower fluorescence intensity due to PCR inhibition), quantification by ddPCR depends on end-point droplet fluorescence which remains higher than the background fluorescence readings. Biological samples including respiratory specimens are often complex and contain PCR inhibitors; therefore, the robust and resistant nature of ddPCR against such inhibitors may be useful in the clinical setting alleviating cost and inconsistent recovery efficiency associated with DNA purification procedures. ddPCR, however, is not fully immune to PCR inhibition, and where severe inhibition exists, ddPCR can experience “total molecular drop-out” where the target remains unamplified [44].

ddPCR permits multiplexing, an additional advantage where clinical material is scarce.

The majority of available *Aspergillus*-related diagnostic kits do not specifically detect *A. fumigatus* and *A. terreus* in biological samples and usually consist of primers and probes that employ a pan- *Aspergillus* approach which cross-react across several *Aspergillus* species. While duplexing is more challenging in a TaqMan qPCR approach, largely due to substrate competition and the need for internal positive controls, simultaneous measurements of *A. fumigatus* and *A. terreus* are possible with ddPCR due to the individual amplification approach of the generated droplets. ddPCR duplexing while reducing labor, improving logistic arrangements and optimizing data quality through limiting accumulated pipetting errors and the lack of a need for standards does come with the added costs of reagents and consumables unique to ddPCR.

One key drawback, however, of ddPCR is its poor performance with high abundance samples containing >10^5^ gene copy numbers. This is due to the partitioning aspect of this technology, the number of droplets generated and the Poisson distribution algorithm employed for the accurate determination of the number of DNA copies per sample [11]. Therefore, in such circumstances, sample dilution may be required, which poses an additional experimental variability, or one can simply revert to TaqMan qPCR. Sample processing, droplet generation, thermal cycling and droplet analysis further adds additional processing time (~2 h) to the overall process compared to qPCR. The balance between achieving good sensitivity and high accuracy for any test is critically important. Sensitivity is the proportion of true positives that are correctly identified by a diagnostic test while accuracy is the overall proportion of true results, whether true positive or true negative. In our work, sensitivity was high for both qPCR and ddPCR approaches but highest using the latter. Accuracy also remained high for both the healthy and diseased specimens tested in this work however again was highest using the ddPCR approach. Therefore, ddPCR appears to be both highly sensitive and accurate, likely explained by the reaction being performed at the individual droplet level as compared to qPCR where competition for the substrate reagents occurs particularly in duplex assays and these reactions remain subject to PCR inhibition.

While several groups internationally, including ours, have developed qPCR protocols to detect *Aspergillus* species in biological samples, emerging technologies such as ddPCR offer an alternate, highly sensitive and accurate quantification for samples with ultra-low microbial (fungal) burdens such as that of the airway where detection, even in small amounts has an important clinical consequence and may be potentially missed by qPCR [1,2,3,4,5,7,45]. Attributes such as that offered by ddPCR may be useful in the current era, where microbiomes including the fungal mycobiome, are taking on greater importance and relevance in understanding pathogenesis, disease course and their consequence across a range of respiratory disease states [1,3,27,30,41,42,46,47]. Going beyond fungi, the promise of ddPCR will likely extend across a wide range of microorganisms, organ systems and human disease if it is appropriately applied to the right specimen, in the right setting and to resolve a specific clinical question.

## 4. Materials and Methods

### 4.1. Design of Primers and Probes:

#### Aspergillus

Species-specific assays were designed employing the NCBI primer-blast software using the 18S ITS sequence of Aspergillus fumigatus (accession number NR_121481) and Aspergillus terreus (accession number NR_131276). Primers and probes annealing to sequences of the respective target Aspergillus species were designed to ensure selectivity for no other fungal genera. To ensure that no non-specific interactions between primers and probes occur (for duplex PCR), the Multiple Primer Analyzer (https://www.thermofisher.com) software was employed to exclude the presence of primer-dimers. Cloning of Aspergillus ITS sequences was performed using pan-Aspergillus PCR primers. Primer sequences were derived by homology alignment of the conserved regions between the 18S rRNA genes of the 4 most clinically isolated Aspergillus species (A. fumigatus, A. terreus, A. flavus and A. niger). All primers and probes were synthesized by Bio Basic Asia Pacific Pte Ltd. (Singapore). All sequences of the primers and probes used in this study are shown in Table 4 and specific locations of RT-PCR assay targets in Appendix A.

### 4.2. Growth and Harvesting of Fungal Cultures

Fungal conidia were isolated from *A. fumigatus* and *A. terreus,* respectively, after growth on Sabouraud agar for 2–7 days, dependent on growth rate. Conidia were harvested by washing the culture with phosphate-buffered saline (PBS) containing 0.05% Tween 20. Conidia were then separated from hyphal fungal elements by filtration through sterile gauze. Following centrifugation at 2000× *g* for 2 min, conidia were washed twice (with sterile PBS) and resuspended in PBS. Conidia were then used for direct DNA extraction as positive-control material and as sources for cloning experiments.

### 4.3. Cloning of A. Fumigatus and A. Terreus ITS

*A. fumigatus* and *A. terreus* ITS plasmids were employed for absolute quantification and cloned from the conidial extracted DNA using the designed pan-*Aspergillus* ITS primers (Table 4). PCR was carried out using the KAPA HiFi HotStart ReadyMix (Roche, Basel, Switzerland) which contained 12.5 µL of 2X KAPA HiFi HotStart ReadyMix, 0.5 µM of each primer and 10 ng of *Aspergillus* DNA in a final volume of 25 µL. Cycling conditions were as follows: 1 cycle at 95 °C for 3 min, 30 cycles at 98 °C for 20 s, 60 °C for 15 s and 72 °C for 15 s with a final cycle at 72 °C for 1 min. PCR products were then gel purified using the Qiagen gel purification kit according to the manufacturer’s instructions. Adenosine overhangs were next added to the PCR product using the 2× PCR Master Mix (Thermo Fisher Scientific, Waltham, MA, United States) with a final volume of 10 µL and cycling at 72 °C for 15 min. PCR products were next cloned using the TOPO™ TA Cloning™ Kit for Sequencing (Invitrogen, Waltham, MA, United States) with One Shot™ TOP10 Chemically Competent E. coli according to the manufacturer’s instructions. Plasmids were then extracted with the Qiagen miniprep kit (Qiagen, Hilden, Germany) according to the manufacturer’s instructions and plasmid sequences verified through sequencing (Bio Basic Asia Pacific Pte Ltd., Singapore).

### 4.4. Study Population

Stable patients (age 21 years or above) with COPD and bronchiectasis were recruited from Singapore General Hospital between March 2016 and July 2018 (Table 1). Stability was defined as the absence of acute symptomatic deterioration (or exacerbation) and/or infection in the preceding four-week period prior to study recruitment. Chronic respiratory disease states were diagnosed based on established international guidelines and are further described below. A separate cohort of non-diseased subjects aged >21 years old with no underlying respiratory disease or other medical history, with normal pulmonary function were recruited through an established program at Nanyang Technological University, Singapore. COPD patients were diagnosed based on the Global Initiative for COPD (GOLD) guidelines and were all > 40 years old, had a smoking history of > 10 packs a year and abnormal lung function defined as forced expiratory volume in one second (FEV1)/forced vital capacity (FVC) ratio < 0.7 and FEV1 < 80% predicted with symptoms defined as presence of cough and/or sputum or breathlessness [48]. Bronchiectasis was confirmed by high-resolution computed tomography (HRCT) and the presence of two or more clinical symptoms of cough, mucopurulent sputum production and shortness of breath and the exclusion of other predominant chronic respiratory disease states [49,50,51]. Patients were excluded if they were pregnant or breastfeeding, had active mycobacterial disease, or were on chemotherapy in any form. Table 5 summarizes the patient demographics.

### 4.5. Ethical Approval

All subjects gave their informed consent for study inclusion. The study was conducted in accordance with the Declaration of Helsinki, and the protocol was approved by the Ethics Committee of NTU and all participating hospitals as follows: CIRB 2016/2715, 2016/2628 and CIRB 2016/2073 (Singapore General Hospital), IRB-2017-03-013, IRB-2016-10-057 and IRB-2016-01-031 (Nanyang Technological University, Singapore).

### 4.6. Specimen (Sputum) Collection and DNA Extraction

Spontaneously expectorated “representative” sputum from a deep cough was collected and examined [52]. After weighing, the sputum was added to an equal volume of Sputasol (Thermo Fisher Scientific, Waltham, MA, United States) containing 0.1% dithiothreitol and incubated for 15 min at 37 °C. Two volumes of RNAlater (Sigma-Aldrich, St. Louis, MO, United States) were added and samples homogenized before DNA extraction [35]. Sputum DNA was extracted using methods as previously described [35]. Briefly, homogenized sputum in RNAlater were centrifuged (13,000 rpm for 10 min) and pellets resuspended in 500 µL sterile PBS (GE Lifesciences, Marlborough, MA, United States). After transfer to sterile bead mill tubes (VWR) containing acid washed 1 mm sterile glass beads (Sigma-Aldrich, St. Louis, MO, United States), DNA extraction was performed using the Roche High Pure polymerase chain reaction (PCR) Template Preparation Kit (Roche, Basel, Switzerland) according to the manufacturer’s instructions. The ratio of absorbance at 260 nm and 280 nm by Nanodrop was used to assess the purity of DNA. A ratio of ~1.8 is generally accepted as “pure” DNA.

### 4.7. Quantitative-PCR (qPCR) and Digital Droplet PCR (ddPCR) Detection of Aspergillus Species

The presence of *A. fumigatus* and *A. terreus* from human airway specimens were assessed by a probe-based qPCR assay as previously described [1]. An *A. fumigatus* probe (tagged with a 5′ 6-carboxyfluorescein (FAM) reporter dye and a 3′ Black Hole Quencher 1 (BHQ1) quencher) and *A. terreus* probe (tagged with a 5′ HEX reporter dye and a 3′ Black Hole Quencher 1 quencher, Bio Basic Asia Pacific, Singapore) were used. qPCR assays with C_qs_ values of <40 were considered positive. The C_qs_ cut-off of 40 was used in line with our prior published work with *A. fumigatus* and *terreus* species which is previously validated through microbial sequencing approaches [1]. Each TaqMan reaction mix contained 1× PCR master mix (PrimeTime Gene Expression Mastermix, Integrated DNA Technologies Pte. Ltd., Singapore), 900nM of forward and reverse primers each, 250 nM of probe, 1 µL of DNA template; and 2.5 µL of internal positive control (Applied Biosystems^®^ TaqMan^®^ Exogenous Internal Positive Control) to make a final volume of 25 µL. Inclusion of the internal positive control allowed assessment of PCR inhibitors in the DNA extract for each respective airway specimen including the no template control (NTC). Reactions were performed with the Applied Biosystems^®^ MicroAmp^®^ Fast Optical 96-Well Reaction Plate on a StepOne™ Real-Time PCR System (Applied Biosystems, Foster City, CA, United States) under the following conditions: 95 °C for 3 min, followed by 40 cycles of denaturation at 95 °C for 5 s and annealing/extension at 60 °C for 30 s. The same assay was adapted for ddPCR (Bio-Rad QX200, Hercules, CA, United States) excluding the IPS control. For ddPCR, each 25 µL reaction setup contained droplet PCR Supermix (Bio-Rad, Hercules, CA, United States), 900 nmol of each primer, 250 nmol of the probe and 1 µL of sample DNA. The reaction volume was then mixed with droplet generation oil (20 µL mixture with 70 µL oil) via microfluidics using the Bio-Rad Automated Droplet Generator (Bio-Rad, Hercules, CA, United States). The water-in-oil droplets were transferred to 96-well PCR plates and heat-sealed to be run on the Bio-Rad C1000 (Bio-Rad, Hercules, CA, United States) thermocycler (ramping speed at 2 °C/s) for PCR amplification using the following conditions: activation for 10 min at 95 °C, followed by 40 cycles of denaturation for 30 s at 94 °C and annealing/extension for 60 s at 60 °C, followed by a 10 min hold step at 98 °C. On completion, automated measurements of fluorescence for each droplet were determined using the QX200 Droplet Reader (Bio-Rad, Hercules, CA, United States) with the RED (rare event detection) setting. No standard curve was required for ddPCR experiments and the droplets quantified using the Bio-Rad QuantiSoft^TM^ software version 1.7 (Bio-Rad, Hercules, CA, United States). Two replicates per sample were performed and a threshold cut-off of 20,000 employed (based on optimization experiments which accurately separated positive from negative droplets). For both protocols, no template controls comprising DNase- and RNase-free water were included. To ensure a thorough assessment of false-negative results from either qPCR or ddPCR for all included clinical specimens (*n* = 20), any samples showing no amplification or an amplification discrepancy between qPCR and ddPCR was re-tested for confirmation. All included healthy controls (*n* = 4) were known to be *Aspergillus* negative and all included samples from patients with chronic respiratory disease (*n* = 16) known to be *Aspergillus* positive by 18S ITS sequencing hence a detailed evaluation of false-positive rates was limited. All results from qPCR and ddPCR assays were compared to these samples of known *Aspergillus* status. In compliance with established guidelines, supplemental information (Supplementary Table S1) is provided in regards to methodologies used in this study.

### 4.8. Data Analysis (qPCR)

For TaqMan qPCR, data was analyzed using StepOne^TM^ software (version 2.3, ABI Biosystems, Foster City, CA, United States). Fluorescence thresholds for *A. fumigatus* and *A. terreus* were set at 0.1 to obtain C_qs_ values. Quantification of fungal burden in clinical specimens was based on the derived standard curves.

### 4.9. Data Analysis (ddPCR)

ddPCR data was analyzed using QuantaSoft™ software (version 1.7 Bio-Rad, Hercules, CA, United States) following the manufacturer’s recommendations. Total droplet number was determined and only wells containing > 10,000 droplets accepted for analysis. Fluorescence thresholds were set to approximately 2SDs (~2000 fluorescence units) above background fluorescence measurement of negative droplets from NTC wells. Target concentrations in copy number per ml reaction were then automatically calculated by QuantaSoft software. Wells with >1 positive droplet were considered positive. The copy number per µL reaction (x) was then determined by multiplication by a factor of 40 as the final reaction mix was 40 µL.

## 5. Conclusions

ddPCR represents an alternate methodology for the detection and quantification of *Aspergillus* species in the human airway. Here, we evaluate its sensitivity for quantifying airway *Aspergillus* burden, for *A. fumigatus* and *A. terreus* against a standard TaqMan qPCR approach employing human airway specimens from patients with chronic respiratory disease. While both approaches (standard TaqMan qPCR and ddPCR) can detect these *Aspergilli* (*A. fumigatus* and *A. terreus*), ddPCR importantly demonstrates better sensitivity and greater resistance to PCR inhibition for *A. terreus* particularly when present at low abundance. Considering the emerging importance of *A. terreus* species in chronic respiratory disease states such as bronchiectasis, ddPCR represents a useful, viable and reliable alternative to qPCR in such patients.

## Figures and Tables

**Table 1:** Summary of comparison between qPCR and ddPCR.

	Real-Time Quantitative PCR	Droplet Digital PCR
Overview	Measures PCR amplification as it occurs	Measures the fraction of positive and negative replicates to determine absolute copies
		
Quantitative measurement	Yes	Yes
		
Methods of data collection	Data is collected during the exponential growth (log) phase of the PCR reaction when the quantity of the PCR product is directly proportional to the amount of template nucleic acid	PCR reaction is partitioned into thousands of individual real-time PCR reactions prior to amplification and data only acquired at the end-point
		
Method of calculation	Targets with unknown quantity are compared to a standard curve with known quantities and a value extrapolated	The fraction of positive and negative reactions are used to generate an absolute value for the exact number of target molecules within a sample according to a Poisson distribution statistical algorithm
		
Relative or Absolute quantification	Both, however a standard curve with known absolute quantities of the target is needed for absolute quantification	Absolute, ddPCR provides an absolute count of target DNA copies per input sample without the need for standard curves
		
Reproducibility	Moderate as quantification can be influenced by PCR efficiency bias between runs	High
		
Single-plex or multiplex	Both, up to 5-plex	Both, up to 5-plex [13]
		
Sensitivity	Moderate: with detection limit from 1 to 10	High: with a detection limit as low as 1 in 2000
		
Other advantages	No post-PCR processing Wide choice in detection chemistry Reaction volume can be optimized to allow for flexible running costs Increased dynamic range of detection	References or standard curves not required Highly tolerant to inhibitors Capable of analyzing complex mixtures Desired precision can be achieved by increasing the total number of PCR replicates
		
Applications	Quantification of gene expression Pathogen detection microRNA analysis microarray verification SNP genotyping Quality control and assay validation siRNA/RNAi experiments	Absolute quantification of microbial load e.g., bacteria, viruses and fungi Absolute quantification of nucleic acid standards Absolute quantification of next-generation sequencing libraries Absolute quantification of gene expression Rare allele detection Mutation screening

**Table 2:** Table illustrating plasmid concentration, expected absolute plasmid copy number and corresponding 40 minus Cqs results from TaqMan qPCR. C_qs_—quantification cycle, ng/mL—nanogram per milliliter, fg/mL—femtogram per milliliter.

Standards	*Aspergillus fumigatus*			*Aspergillus terreus*		
Plasmid Concentration, ng/mL (fg/mL)	40 Minus C_qs_	Standard Error	Absolute Copy Number	40 Minus Cqs	Standard Error	Absolute Copy Number
0.001 (1000 fg/mL)	20.46	1.51	216,516.79	16.53	0.13	215,988.54
0.0001 (100 fg/mL)	17.18	1.63	21,651.68	13.82	0.5	21,598.854
0.00001 (10 fg/mL)	11.69	0.91	2165.17	10.92	0.19	2159.8854
0.000001(1 fg/mL)	10.22	0.06	216.52	3.45	0.85	215.98854
0.0000001 (0.1 fg/mL)	6.77	0.07	21.65	1.4	1.98	21.598854

**Table 3:** Table illustrating absolute copy numbers of *A. fumigatus* and *A. terreus* derived by ddPCR.

	*Aspergillus fumigatus*	*Aspergillus terreus*
Plasmid Concentration, ng/mL (fg/mL)	Absolute Copy Number	Absolute Copy Number
0.001 (1000 fg/mL)	82,480	80,900
0.0001 (100 fg/mL)	5140	7940
0.00001 (10 fg/mL)	438	886
0.000001 (1 fg/mL)	68	172
0.0000001 (0.1fg/mL)	20	40

**Table 4:** Summary of all primers and probes used in this study. 6-FAM: 6-Carboxyfluorescein, BHQ1: Black Hole Quencher-1, ITS: internal transcribed spacer, Mod: modification.

Oligo Name	Sequence (5′ to 3′)	Amplicon	5′ Mod	3′ Mod
*Aspergillus fumigatus* forward primer	TTGTCACCTGCTCTGTAGGC	83 bp	None	None
*Aspergillus fumigatus* reverse primer	TCCCTACCTGATCCGAGGTC		None	None
*Aspergillus fumigatus* probe	CCGGCGCCAGCCGACACCCA		6-FAM	BHQ1
*Aspergillus terreus* forward primer	CATTACCGAGTGCGGGTCTTTA	70 bp	None	None
*Aspergillus terreus* reverse primer	CCCGCCGAAGCAACAAG		None	None
*Aspergillus terreus* probe	CCCAACCTCCCACCCGTGACTATTG		HEX	BHQ1
Pan *Aspergillus* ITS forward primer	CGGAAGGATCATTACCGAGT	Unknown	None	None
Pan *Aspergillus* ITS reverse primer	CCTACCTGATCCGAGGTCAA		None	None

**Table 5:** Demographics of the study population. Data are presented as median and interquartile ranges (IQR) or n (percentage; %). COPD: chronic obstructive pulmonary disease, Post BD: post bronchodilator; BMI: body mass index; BSI: Bronchiectasis Severity Index.

Characteristics	Non-Diseased (Healthy, *n* = 4)	COPD (*n* = 8)	Bronchiectasis (*n* = 8)
Age (years): Median (IQR)	64 (63–66)	71 (68–74)	67 (58–74)
Gender (male): n (%)	1 (25%)	8 (100%)	4 (50%)
BMI (kg/m^2^): Median (IQR)	23 (21.3–24.6)	24.3 (22.5–28.6)	18.3 (16.8–20.5)
Smoking history: n (%)			
Never–smoker	4 (100%)	0 (0)	7 (87.5%)
Current smoker	0 (0%)	3 (37.5%)	0 (0%)
Ex-smoker	0 (0%)	5 (62.5%)	1 (12.5%)
COPD assessment test (CAT): Median IQR)	NA	15 (7.3–24.5)	NA
Post BD FEV1 (% predicted): Medium (IQR)	NA	37.5 (32.8–38)	57.5 (44–80)
Post BD FEV1/FVC (% predicted): Medium (IQR)	NA	44.5 (39.5–49.8)	79 (78–81)
BSI score	NA	NA	8 (6.6–16)
No. of exacerbations in year preceding recruitment: Median (IQR)	NA	1 (1–1)	3 (0–4)

## Supplementary Materials

The following are available online at https://www.mdpi.com/1422-0067/21/9/3043/s1, Table S1: MIQE Guidelines: Essential Information Checklist

## Abbreviations

%	percentage
+ve	positive
<	less than
>	greater than
±	plus/minus
*∆Rn*	delta normalized reporter value
°C	degrees Celsius
µL	microliter
µM	micro-molar
6-FAM	6-carboxyfluorescein
Asp	*Aspergillus*
BHQ1	Black Hole Quencher 1
BLAST	Basic Local Alignment Search Tool
BMI	body mass index
B	bronchiectasis
BSI	Bronchiectasis Severity Index
CAT	COPD assessment test
COPDCF	chronic obstructive pulmonary diseaseCystic Fibrosis
Cqs	Quantification cycle
ddPCR	droplet digital PCR
DNA	deoxyribonucleic acid
FEV1	forced expiratory volume in the first second
Fg	femtogram
FVC	forced vital capacity
*g*	gravitational force
HRCT	high-resolution computed tomography
IPC	internal positive control
IQR	interquartile range
ITS	internal transcribed spacer
mins	Minutes
Mod	modification
N	sample size
next-gen	next-generation
ng	nanogram
nM	nano-molar
No.	Number
PBS	phosphate-buffered saline
pg	picogram
Post BD	post bronchodilator
Pte Ltd.	Private Limited
qPCR	quantitative polymerase chain reaction
RNAi	RNA interference
rpm	rounds per minute
rRNA	ribosomal ribonucleic acid
Secs	Seconds
siRNA	small interfering ribonucleic acid
SNP	single nucleotide polymorphism
*Taq*	*Thermus aquaticus*
-ve	Negative

## Appendix A

Alignment of four clinically important *Aspergillus* species. TaqMan primers (blue) and probes (red) are shown by their respective coloration. Pan-*Aspergillus* primers are indicated by boxes.
